# Integrated analysis of carotenoid metabolites and transcriptome identifies key genes controlling carotenoid compositions and content in sweetpotato tuberous roots (*Ipomoea batatas* L.)

**DOI:** 10.3389/fpls.2022.993682

**Published:** 2022-10-20

**Authors:** Ruixue Jia, Rong Zhang, Sunil S. Gangurde, Chaochen Tang, Bingzhi Jiang, Guilan Li, Zhangying Wang

**Affiliations:** ^1^ College of Agronomy and Biotechnology, Hebei Normal University of Science and Technology, Changli, China; ^2^ Crops Research Institute, Guangdong Academy of Agricultural Sciences & Key Laboratory of Crop Genetic Improvement of Guangdong Province, Guangzhou, China; ^3^ International Crops Research Institute for the Semi-Arid Tropics (ICRISAT), Hyderabad, India; ^4^ Crop Protection and Management Research Unit, USDA-ARS, Tifton, GA, United States; ^5^ Department of Plant Pathology, University of Georgia, Tifton, GA, United States

**Keywords:** sweetpotato, coloration, key genes, transcription factor, WGCNA

## Abstract

Sweetpotato (*Ipomoea batatas* L.) with different depths of yellow color contains different compositions of carotenoids, which are beneficial for human health. In this study, we performed an integrated analysis of metabolomic and transcriptomic to identify key genes playing a major role in carotenoid coloration in sweetpotato tuberous roots. Herein, 14 carotenoids were identified in five sweetpotatoes. Orange-red and orange cultivars were dominated by β-carotene (385.33 μg/g and 85.07 μg/g), yellow cultivar had a high β-cryptoxanthin (11.23 μg/g), light-yellow cultivar was rich in zeaxanthin (5.12 μg/g), whereas lutein (3.34 μg/g) was the main carotenoid in white cultivar. Furthermore, 27 differentially expressed genes involved in carotenoid metabolism were identified based on comparative transcriptome. Weighted gene co-expression network analysis identified 15 transcription factors highly associated with carotenoid content in sweetpotatoes. These results provide valuable information for revealing the regulatory mechanism of carotenoid metabolism in different-colored sweetpotato tuberous roots.

## Introduction

Sweetpotato(*Ipomoea batatas* L.) ranks the sixth largest food crop in the world and has played an important role as a famine-relief crop in its long history (CIP, 2020). Recently, sweetpotato has been re-evaluated as a health-promoting food and successfully demonstrated to tackle vitamin A deficiency with biofortification in sub-Saharan Africa due to its high accumulation of β-carotene (World Food Prize Foundation, 2016). In Asia, yellow-fleshed cultivars are popular for table use and processed products. The flesh color of the sweetpotato has become one of the most important targets in breeding programs. Sweetpotato cultivars show various flesh colors such as white, yellow, orange and purple due to the accumulation of different contents of pigments. Among them, the yellow and orange flesh colors are determined by the carotenoid content and composition (Ishiguro et al., 2010). In addition to being an excellent source of provitamin A in humans, carotenoids also have the function of antioxidant activity, anticancer activity, reducing the progression of age-related macular eye disease in human health (Eggersdorfer and Wyss, 2018; Fraser and Bramley, 2004; Islam et al., 2016).

Due to the visibility and importance of carotenoid in plants and humans, its biosynthesis pathway and the associated biosynthetic enzymes have been thoroughly investigated (Fraser and Bramley, 2004; Sajilata et al., 2008). Geranylgeranyl diphosphate (*GGPP*) is a common precursor of carotenoids, ubiquinones, abscisic acid, tocopherols, gibberellin and chlorophyll (Beck et al., 2013). Carotenoid biosynthesis pathway consists of two processes to produce two different kinds of carotenoids. First, the *GGPP* is converted into a group of carotenoids, containing only carbon and hydrogen called as carotenes by a series of enzymes. Further, the carotenes are turn converted into other group of carotenoids, containing one or more oxygen atoms called as xanthophylls (Armstrong, 1997). Precursor *GGPP* produces colorless phytoene through condensation catalyzed by 15-cis-phytoene synthase (*PSY*) and the phytoene produces red lycopene through a series of enzymes (*PDS*, 15-cis-phytoene desaturase; *Z-ISO*, 15-cis-ζ-carotene isomerase; *ZDS*, ζ-carotene desaturase; *CRTISO*, prolycopene isomerase). Lycopene is the critical substance in the carotenoid biosynthesis pathway, which is then differentiates into two branches: γ-carotene and δ-carotene. In the δ-carotene branch, lutein is finally produced by lycopene ϵ-cyclase (*LCYE*), lycopene β-cyclase (*LCYB*), and carotenoid ϵ-hydroxylase (CYP97C1) and β-carotene hydroxylase (*CHYB*). In the γ-carotene branch, the carotenoids synthesized with the help of lycopene β-cyclase (*LCYB*), β-carotene hydroxylase (*CHYB*), zeaxanthin epoxidase (*ZEP*), violaxanthin de-epoxidase (*VDE*) (Liu et al., 2020; Suematsu et al., 2020; Xu et al., 2021; KEGG, 2022).

As mentioned above, cultivated sweetpotatoes have the ability to synthesize various kinds of carotenoids in tuberous root, which are responsible for the vivid flesh colors. The carotenoids in the tuberous root of sweetpotato include β-carotene, β-cryptoxanthin, zeaxanthin, violaxanthin and other carotenoids (Ishiguro et al., 2010; Kim et al., 2012; Kim et al., 2013; Kim et al., 2014). Ishiguro et al. (Ishiguro et al., 2010) analyzed the different colors of eight sweetpotato cultivars and breeding lines, and showed that the main carotenoids in five different yellow-fleshed sweetpotato are β-carotene 5,8;5′,8′-diepoxide (32–51%) and β-cryptoxanthin 5,8-epoxide (11–30%), and β-carotene (< 10%). The main carotenoid in orange-fleshed sweetpotato is β-carotene (80–92%), whereas other carotenoids constitute less than 2% of the total in three different cultivars (Ishiguro et al., 2010). Some genes encoding enzymes in the carotenoid metabolic pathway, including *PSY*, *PDS*, *ZDS*, *CRTISO*, *LCYB*, *LCYE*, *CHYB*, ZEP, 9-cis-epoxycarotenoid dioxygenase (*NCED*), and carotenoid cleavage dioxygenases (*CCD*), have previously been cloned and characterized in sweetpotato (Kim et al., 2013; Kang et al., 2017; Kang et al., 2018; Suematsu et al., 2020). It is reported that silencing of *CHYB*, *LCYB*, or *LCYE* using RNAi increases carotenoid accumulation and resistance to abiotic stress such as heat, drought, and salt in transgenic sweetpotato plants and calli (Kim et al., 2012; Kim et al., 2013; Kim et al., 2014; Kang et al., 2017; Kang et al., 2018; Ke et al., 2019). Despite some genes and transcription factors related to carotenoid synthesis have been cloned in sweetpotato, the regulatory network controlling the variation in carotenoid content and composition is not well characterized, and rare regulators have been reported based on comparative transcriptome and co-expression network analysis.

In the present study, liquid chromatography tandem mass spectrometry (LC–MS/MS) was used to detect and quantify carotenoids in five sweetpotato cultivars with different flesh colors. At the same time, the differently expressed genes involved in carotenoid metabolism were identified by using transcriptional data in these five cultivars. In addition, co-expression gene modules were obtained, and key transcription factors related to carotenoid metabolism were also screened through weighted gene co-expression network analysis (*WGCNA*). This work not only clarified the difference of carotenoid content and composition in tuberous roots of sweetpotato with different colors, but also provided important insights into the regulatory network of carotenoid metabolism in sweetpotato.

## Materials and methods

### Plant materials

Five sweetpotato cultivars with different flesh colors were used for metabolome and transcriptome analysis. The cultivars included, Shangshu 19 with white flesh (W), Okinawa-No.100 with light-yellow flesh (LY), Jieshu 95-16 with yellow flesh (Y), Guangshu 87 with orange flesh (O), and Pushu 32 with orange-red flesh (OR). All of the cultivars were planted using the standard production practices at the Baiyun experimental station (23°23′N, 113°26′E, 20 m above sea level) at Guangdong Academy of Agricultural Sciences, Guangzhou, China. Furthermore, samples were harvested in triplicates, 10 medium-sized tuberous roots from each cultivar were selected and divided into two parts. One part was used for color measurement, and the other part was cut into small pieces, mixed well, frozen in liquid nitrogen immediately and stored at -80°C for further metabolite extraction, transcriptome sequencing, and real-time PCR analysis, with three replicates for each sweetpotato cultivar.

### Color measurement

The samples for color measurement were cut in half crosswise, then separately sliced into cross section with 1 cm thickness. The cross-sections of two pieces in the middle of each tuberous root were scanned using an A3 Unis Scanner (Uniscan^®^ M1 Plus, UNIS, China), and the International Commission on Illumination CIE 1976 (L*, a*, b*) color space values were measured using a software of Tomato Analyzer 3.0 (Rodríguez et al., 2010). Before measuring the color, the software was standardized with a color calibration card. The average of the three corresponding readings of each cultivar was considered and analyzed statistically as the final values. Results were described as L* (brightness or lightness, positive towards white and negative towards black), a* (red-green, positive towards red and negative towards green), b* (yellow-blue, positive towards yellow and negative towards blue), H° (hue angle, calculated from the arctangent of b*/a*, 0° or 360° towards red, 90° towards yellow, 180° towards green, 270° towards blue), and C* (chroma, calculated as $\sqrt{(a^{*2}+b^{*2\;}}$ ) (Mcguire, 1992).

### Carotenoid extraction

The carotenoid was extracted by the method described by Ma et al. (Ma et al., 2017) with slight modifications. The flesh samples of sweetpotato were freeze-dried in a freeze dryer (Xinzhi 16-0350, Ningbo, China) for 64 h with a minimum temperature of – 50°C. Then lyophilized flesh samples were homogenized and powdered in a mill using a grinder with 30 Hz for 1 min (MM 400, Retsch, Germany). 50 mg freeze dried powder was extracted with a solvent containing n-hexane: acetone: ethanol (1:1:1, V/V/V), 0.01% BHT and internal standard were added. The extract was vortexed for 20 min at room temperature, centrifuged and the supernatant was collected. The residue was re-extracted repeating the above step. The extract was evaporated to dryness under nitrogen gas stream and re-suspended in mixed solution of methanol: methyltert-butyl ether (1:1, V/V). The solution was filtered through a 0.22 μm filter for further LC-MS analysis.

### Identification and quantification of carotenoid

The sample extracts were measured using a UPLC-APCI-MS/MS system (UPLC, ExionLC™ AD, MS, Applied Biosystems 6500 Triple Quadrupole) with a YMC C30 column (3 μm, 100 mm × 2.0 mm). Mobile phases were MeCN-MeOH (3:1, V/V) containing 0.01% BHT and 0.1% formic acid (eluent A) and 100% MTBE containing 0.01% BHT (eluent B), and methanol was used as the probe wash. The flow rate was set to 0.8 mL/min, the column temperature was set at 28°C, and the injection volume was set as 2 μL. The gradient programs (eluent A: eluent B) were as follows: 100:0 V/V from 0 min, 30:70 V/V at 5.0 min, 5:95 V/V at 9.0 min, 100:0 V/V at 9.1 min, 100:0 V/V at 11.0 min (Inbaraj et al., 2008; Petry and Mercadante, 2017).

MS/MS detection was performed on AB 6500 Triple Quadrupole LC-MS/MS System, equipped with an atmospheric pressure chemical ionization (APCI) heated nebulizer interface. The APCI source operation parameters were as follows: ion source, APCI +; source temperature, 350°C; curtain gas (CUR) was set at 25.0 psi; Declustering potential (DP) and collision energy (CE) for individual MRM transition was done with further DP and CE optimization. A specific set of MRM transitions were monitored for each period according to the carotenoids eluted within this period.

The carotenoid analysis was performed using the Metware database (MWDB, Wuhan, China) constructed from the standards to qualitatively analyze the mass spectrometry data. We analyzed LC-MS/MS data from the carotenoids using Analyst 1.6.3 software (AB Sciex) with default parameters for automatic identification of changes and integrals in the MRM. Carotenoid retention times and ion pair information were used to modify chromatographic peaks for each carotenoid in different samples (**Figure S1**). Standard curves of different carotenoids were drawn with the concentration ratio of external standard to internal standard as the abscissa and the peak area ratio of chromatographic peak as the ordinate (**Table S1**). Then the ratio of the integral peak area of each carotenoid detected in the samples to the peak area of the internal standard was substituted into the standard curves. Finally, the absolute content of carotenoids in the actual samples was calculated using the following formula: carotenoid content (μg/g) = c*V/1000/m, where c is the concentration value obtained by substituting the integrated peak area ratio of the sample into the standard curve (μg/mL), V is the resuspension volume (μL), and m is the sample weight (g).

### RNA-seq analysis and functional annotation

The total RNA was extracted from frozen samples of five sweetpotato genotypes (three replicates of each). RNA purity, concentration and integrity were analyzed on NanoPhotometer^®^ spectrophotometer (IMPLEN, CA, USA), Qubit^®^ RNA Assay Kit in Qubit^®^2.0 Fluorometer (Life Technologies, CA, USA), and RNA Nano 6000 Assay Kit of the Bioanalyzer 2100 system (Agilent Technologies, CA, USA).

One µg RNA of each sample was used for construction of sequencing libraries. Sequencing libraries were generated using NEBNext^®^ Ultra™ RNA Library Prep Kit for Illumina^®^ (NEB, USA) following manufacturer’s recommendations and index codes were added to attribute sequences to each sample. mRNA was purified from total RNA using poly-T oligo attached magnetic beads. The first and second strand cDNA was synthesized, double-strand cDNA was purified, the adapters were added. In order to select cDNA fragments of preferentially 250~300 bp in length, the library fragments were purified with AMPure XP system (Beckman Coulter, Beverly, USA). Then sequencing libraries were sequenced on an Illumina NovaSeq 6000 system. The quality of sequence data was checked and adapters sequences and low-quality sequences were removed using Fastp with default parameters (Chen et al., 2018). Clean reads were mapped to the reference genome of sweetpotato (Yang et al., 2017) using HISAT2 (Kim et al., 2015). The gene alignment was calculated with FeatureCounts v1.6.2 (Liao et al., 2014), and the FPKM of each gene was calculated based on the gene length. Based on the original count data, differential gene expression between the samples was calculated using DESeq2 v1.22.1 (Varet et al., 2016). The genes with |log_2_ Fold Change| ≥ 1 and False Discovery Rate (FDR)< 0.05 were called differentially expressed genes (DEGs). All transcriptome data have been uploaded to the China National Genomics Data Center (NGDC) database (BIG Sub - BioProject (cncb.ac.cn)), and the BioProject ID is PRJCA008295.

The gene enrichment analysis is performed based on the hypergeometric test. Gene function annotation was performed using four primary databases: Gene Ontology (GO), Kyoto Encyclopedia of Genes and Genomes (KEGG) database, NCBI non-redundant protein sequences (NR) and Swiss-Prot.

### Co-expression networks analysis

To identify the potential candidate genes involved in regulation of carotenoid accumulation, differentially expression genes (DEGs) and variability in carotenoid contents detected in the five cultivars were selected for performing integrative analysis. Weighted gene co-expression networks analysis (WGCNA) was performed in R package WGCNA (Langfelder and Horvath, 2008), based on 18,340 genes with normalized FPKM values and the carotenoid content in different samples. Cytoscape (Otasek et al., 2019) software was used to visualize co-expression network in each module.

### Validation using quantitative real-time polymerase chain reaction

The total RNA was extracted from the flesh of five sweetpotato cultivars according to MiniBEST Plant RNA Extraction Kit (TaKaRa, Beijing, China). The first-strand cDNA was synthesized using TIANGEN FastKing gDNA Dispelling RT SuperMix (TIANGEN, Beijing, China). In this study, we randomly selected five genes from RNA-seq for qRT-PCR analysis, and used the *Ibactin* gene (AY905538) as a reference gene to correct gene expression. In addition, all primers used in this study were designed by Primer 3Plus (Untergasser et al., 2007), and listed in Supplement **Table S2**. Real-time quantitation PCR analysis was performed by Bio-Rad CFX96 system (Hercules, CA, United States) using a SsoFast™ EvaGreen Supermix (Bio-Rad) at temperatures 95°C for 3 min and 40 cycles of 95°C for 15 s, 55°C for 10 s, and 72°C for 30 s. The melting curve, carrying out 61 cycles with 0.5°C increments from 65 to 95°C. The qRT-PCR data was generated in three replicates for each sample, and the quantitative data was analyzed by the 2^-ΔΔCt^ method.

### Statistical analysis

All experiments were performed in three biological replicates in this study. The data of carotenoid contents were expressed as mean ± standard deviations. Statistical significance and Pearson correlation coefficients were analyzed using SPSS v.26.0 (IBM, SPSS Inc., Chicago, IL, USA). Significant differences among five cultivars were calculated using one-way ANOVA followed by Duncan’s multiple way test at *P<* 0.05 level. The heatmaps of gene expression level and Venn diagram were drawn by TBtools (Chen et al., 2020).

## Results

### Color assessment

**Figure 1:**
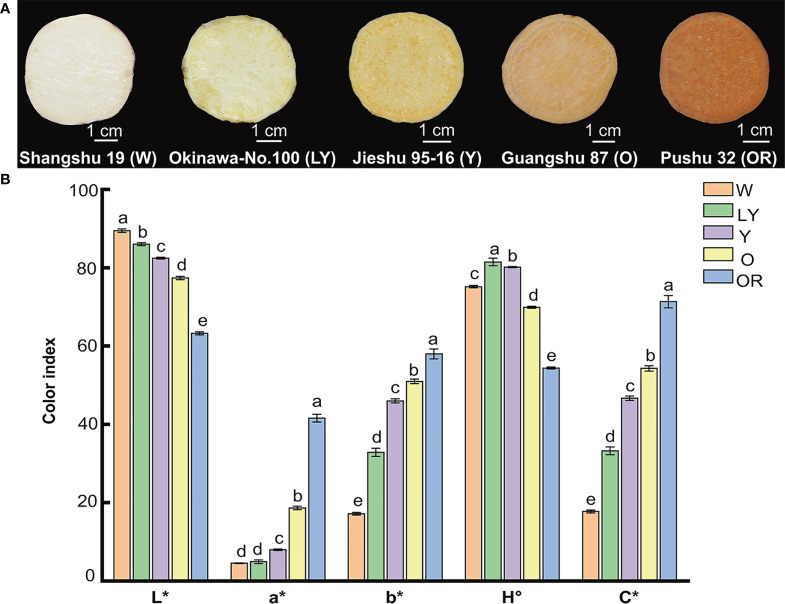
Phenotypic characteristics and color parameters of five sweetpotato fleshes. **(A)** Flesh color of five cultivars. Shangshu 19, Okinawa-No.100, Jieshu 95-16, Guangshu 87, Pushu 32 cultivars were renamed as W, LY, Y, O, OR, respectively. **(B)** The color indexes (L*, a*, b*, H°, C*) of five sweetpotato fleshes. Lowercase letters a, b, c, d and e represent significance at *p* ≤ 0.05. Error bars represent mean ± SD (n = 3).

Five sweetpotato cultivars with different flesh colors: white (W), light-yellow (LY), yellow (Y), orange (O), and orange-red (OR) were used in this study (**Figure 1A**). To understand the color difference between these five cultivars, the color parameters (L*, a*, b*, H°, and C*) of fleshes were measured and illustrated in **Figure 1B**. The parameter values showed significant variation among five sweetpotato cultivars. However, the parameters a*, b*, and C* exhibited a similar tendency across the cultivars, contrary to the trend of parameter L*. Interestingly, we observed that L* value decreased, and a*, b* and C* values increased with the change of flesh color gradient from white to orange-red. The parameter H° of OR cultivar was the lowest, tending to orange-red, while LY cultivar was the largest, tending to yellow. These results were consistent with visual perception. Flesh color in sweetpotato is one of the most important factors which influences the flavor and aroma and therefore attracts consumer attention in the market. Currently, orange-red sweetpotatoes are more popular in the market, which may be largely related to their rich color.

### Composition and content of carotenoids

We estimated the content and composition of carotenoid in five sweetpotatoes of different flesh colors by UPLC-MS/MS (**Table 1**). A total of 14 carotenoids were identified in the non-saponified extracts from five sweetpotato cultivars, of which 11 were identified as free carotenoids or intermediates involved in the carotenoid biosynthetic pathway (including 3 carotenes and 8 xanthophylls) and 3 were identified as carotenoid monoesters. Especially, a total of 9 carotenes namely β-carotene, violaxanthin palmitate, antheraxanthin, zeaxanthin, violaxanthin, neoxanthin, lutein, β-cryptoxanthin, apocarotenal were detected in all five cultivars. Zeaxanthin palmitate was detected in LY, Y, O and OR cultivars, violaxanthin myristate was detected in Y, O and OR cultivars, whereas, γ-carotene, (E/Z)-phytoene and echinenone were detected only in O and OR cultivars.

**Table 1:** Content of carotenoids in five sweetpotato cultivars.

Class	Carotenoid (μg/g DW)	W	LY	Y	O	OR	*P* value	CV (%)
Carotenes	γ-Carotene	–	–	–	0.28 ± 0.02b	2.90 ± 0.53a	< 0.001	187.51
	β-Carotene	0.35 ± 0.02c	0.67 ± 0.11c	3.12 ± 0.30c	85.07 ± 5.40b	385.33 ± 29.14a	< 0.001	162.71
	(E/Z)-Phytoene	–	–	–	2.50 ± 0.20b	10.03 ± 0.81a	< 0.001	160.98
Carotenoid esters	Violaxanthin myristate	–	–	0.09 ± 0.01b	0.22 ± 0.04a	0.19 ± 0.01a	< 0.001	100.00
	Violaxanthin palmitate	0.54 ± 0.07c	0.72 ± 0.05c	0.51 ± 0.04c	1.53 ± 0.12b	2.69 ± 0.42a	< 0.001	73.33
	Zeaxanthin palmitate	–	0.22 ± 0.02c	1.04 ± 0.10b	4.84 ± 0.36a	0.93 ± 0.12b	< 0.001	129.79
Xanthophylls	Antheraxanthin	0.20 ± 0.03d	0.97 ± 0.05b	0.77 ± 0.07c	1.24 ± 0.05a	1.10 ± 0.14b	< 0.001	44.71
	Zeaxanthin	0.26 ± 0.01d	5.12 ± 0.53c	9.29 ± 1.84b	8.84 ± 0.62b	11.63 ± 1.07a	< 0.001	59.89
	Violaxanthin	0.51 ± 0.04c	0.46 ± 0.04c	0.24 ± 0.02c	2.65 ± 0.20b	3.37 ± 0.82a	< 0.001	95.83
	Neoxanthin	0.26 ± 0.03b	0.23 ± 0.03b	0.09 ± 0.01c	0.62 ± 0.10a	0.72 ± 0.09a	< 0.001	66.67
	Lutein	3.34 ± 0.05b	1.19 ± 0.04c	1.07 ± 0.10c	1.43 ± 0.07c	9.28 ± 1.08a	< 0.001	100.00
	β-Cryptoxanthin	0.67 ± 0.06c	2.10 ± 0.17c	11.23 ± 1.04c	35.90 ± 1.71b	139.00 ± 13.75a	< 0.001	143.59
	Apocarotenal	0.01 ± 0.00c	0.01 ± 0.00c	0.01 ± 0.01c	0.05 ± 0.01b	0.28 ± 0.05a	< 0.001	157.14
	Echinenone	–	–	–	0.02 ± 0.00b	0.16 ± 0.02a	< 0.001	150.00

For each carotenoid, different lowercase letters a, b, c, and d in a row indicated significances among means (P< 0.05); SD means standard deviation; CV means coefficient of variance.

The content of 14 carotenoids showed significant variation among the five sweetpotato cultivars (*P*< 0.001). OR cultivar showed highest content of carotenoids among all cultivars except violaxanthin myristate, zeaxanthin palmitate, and antheraxanthin. With the change of color gradient from white to orange-red, the content of β-carotene and β-cryptoxanthin gradually increased. Among the 14 carotenoids, β-carotene was found to be dominant in OR and O cultivars, 385.33 μg/g in OR cultivar and 85.07 μg/g in O cultivar. β-cryptoxanthin and zeaxanthin were enriched in Y and LY cultivars, respectively, while the highest level of carotenoid in W cultivar was lutein (3.34 μg/g). These results indicated that abundant yellow xanthophylls (β-cryptoxanthin, zeaxanthin and lutein) and orange carotenoids (especially β-carotene) may give the yellow and orange colors in sweetpotato fleshes.

### Transcriptomic analysis and function annotation

**Figure 2:**
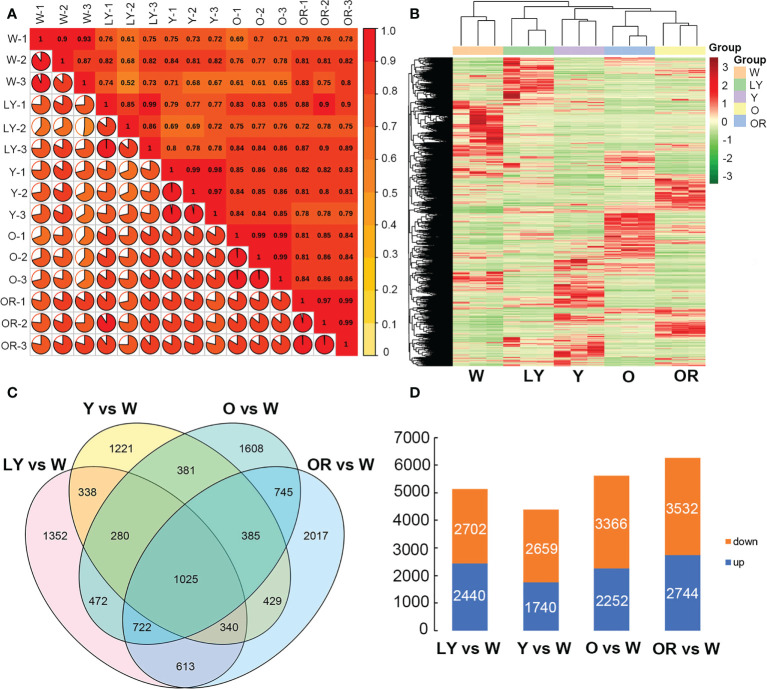
Gene expression profiling of five samples. **(A)** Correlation coefficients between gene expression data sets from three biological duplicates. **(B)** Heatmap illustrates expression pattern of differentially expressed genes in five sweetpotato fleshes. **(C)** Venn diagram showing differentially expressed genes between each pairwise comparison. **(D)** Bar graphs showing the upregulated and downregulated genes in four pairwise combinations.

After filtering the adapter sequences and low quality reads, the average clean reads of 46.26 million, 50.32 million, 46.71 million, 43.39 million and 43.93 million were obtained from W, LY, Y, O, and OR colored fleshes, respectively. The average GC contents were 44.09 ~ 46.21%, and the average Q30 values were 93.58 ~ 94.31% among five cultivars (**Table S3**). All clean reads were mapped to the sweetpotato reference genome, and the mapping rate was ranged from 74.54% to 83.54%. Pearson correlation coefficients were calculated between samples based on the number of fragments per kilobase of exon per million fragments mapped (FPKM) values. The correlation coefficients between the biological replicates of the same cultivar were higher than 0.8 (**Figure 2A**), indicating good reproducibility of the biological repeats. Overall, these results indicated that the quantity and quality of the sequencing data were suitable for the downstream analysis.

A total of 18,340 differentially expressed genes were identified by pairwise comparison between five cultivars based on |log_2_ Fold Change| ≥1 and FDR< 0.05 (**Table S4**). The clustering heatmap of all DEGs was analyzed (**Figure 2B**). A total of 5,142 in LY cultivar, 4,399 in Y cultivar, 5,618 in O cultivar and 6,276 in OR cultivar when compared with W cultivar, and 1,025 DEGs were identified as the common across these combinations (**Figure 2C**). Among these, 2,440, 1,740, 2,252 and 2,744 DEGs were up-regulated in LY *vs.* W, Y *vs.* W, O *vs.* W and OR *vs.* W combinations, respectively (**Tables S5–S8** **;** **Figure 2D** **)**.

KEGG pathway analysis showed that a total of 1,637, 1,534, 1,788 and 2,030 DEGs from the combination of LY *vs.* W, Y *vs.* W, O *vs.* W, OR *vs.* W participated in a total of 130, 131, 131 and 127 KEGG pathways, respectively (**Table S9** and **Figure S2**). Among these pathways, terpenoid backbone biosynthesis and photosynthesis - antenna proteins were significantly upregulated in LY *vs.* W, Y *vs.* W, O *vs.* W combinations. The pathways associated with circadian rhythm-plant and isoflavonoid biosynthesis were highly upregulated in O *vs.* W, OR *vs.* W combinations, while the carotenoid biosynthesis pathway highly enriched in O *vs.* W combination. Based on the GO database, the DEGs were classified into 55, 51, 55, and 57 subcategories from LY *vs.* W, Y *vs.* W, O *vs.* W, OR *vs.* W, respectively (**Table S10** and **Figure S3**). In GO enrichment analysis we observed that secondary metabolite biosynthetic pathways were active in LY/Y/OR *vs.* W combinations. The above results suggested that the circadian rhythm-plant pathway, light system and secondary metabolite biosynthesis might be the key factors responsible for accumulation of pigments in sweetpotato flesh.

### DEGs involved in carotenoid metabolic pathway

**Figure 3:**
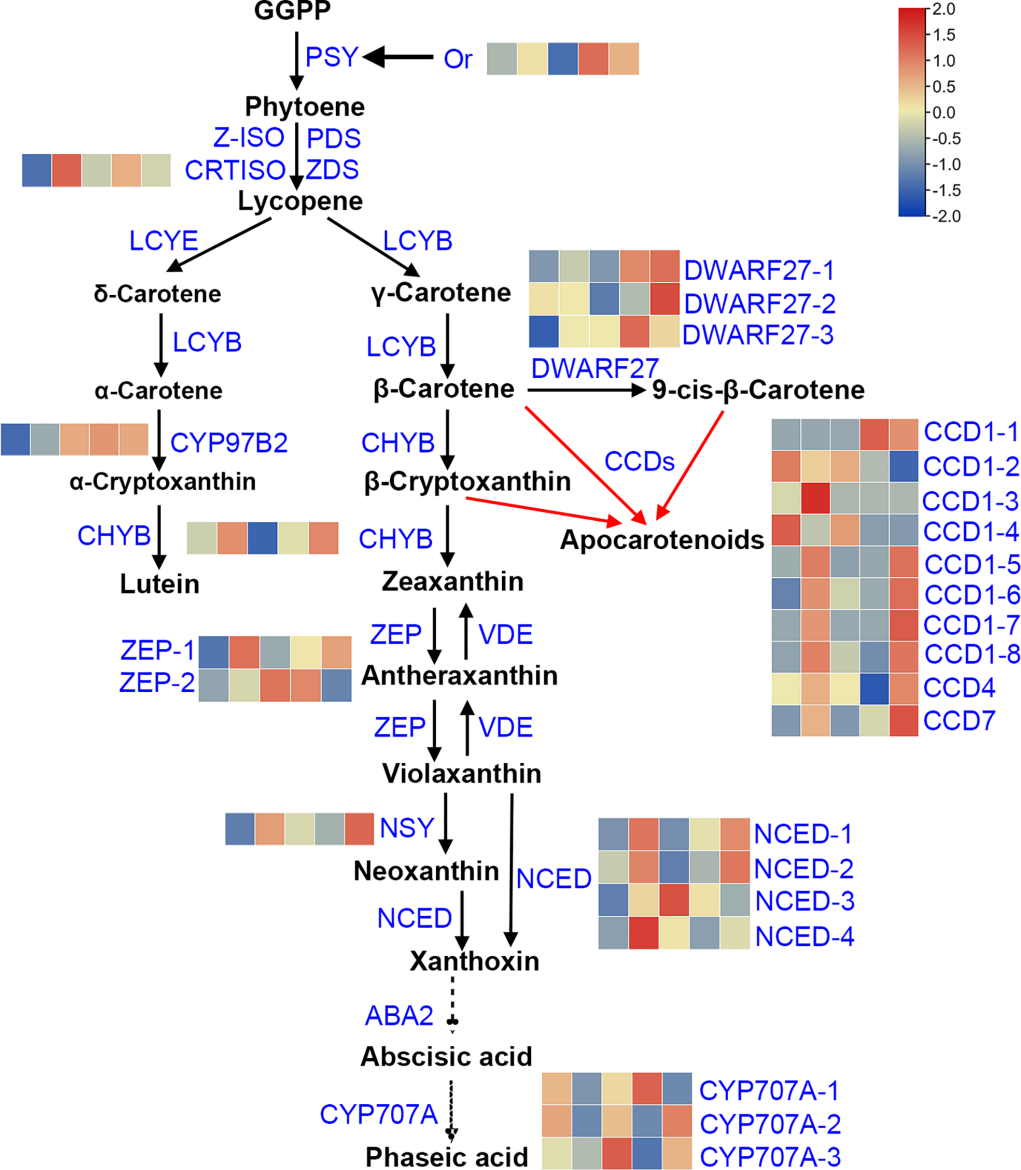
Pathway analysis of carotenoid metabolic in sweetpotato flesh. This pathway was constructed based on the KEGG pathway and references. GGPP, geranylgeranyl diphosphate; PSY, 15-cis-phytoene synthase; PDS, 15-cis-phytoene desaturase; Z-ISO, 15-cis-ζ-carotene isomerase; ZDS, ζ-carotene desaturase; CRTISO, prolycopene isomerase; LCYB, lycopene β-cyclase; LCYE, lycopene ϵ-cyclase; DWARF27, β-carotene isomerase D27; CCD1, carotenoid cleavage dioxygenase 1; CCD4, carotenoid cleavage dioxygenase 4; CCD7, carotenoid cleavage dioxygenase 7; CHYB, β-carotene hydroxylase; CYP97B2, cytochrome P450 97B2-type epsilon-hydroxylase; ZEP, zeaxanthin epoxidase; VDE, violaxanthin de-epoxidase; NSY, neoxanthin synthase; NCED, 9-cis-epoxycarotenoid dioxygenase; ABA2, xanthoxin dehydrogenase; CYP707A, (+)-abscisic acid 8’-hydroxylase. Heatmap of the expression of DEGs involved in carotenoid synthesis pathway, rows and columns represent gene names and samples (from left to right is W, LY, Y, O and OR), respectively.

The flesh color in sweetpotatoes not solely depends on carotenoid content, it is also regulated by the expression level of the intermediate key genes involved in carotenoid biosynthesis and degradation. Further to reveal the mechanism of carotenoid variation in sweetpotato flesh, the DEGs involved in carotenoid metabolic pathway were analyzed. KEGG enrichment analysis and NR annotation showed that, there are 27 structural genes participated in carotenoid metabolic pathway among the four combinations (LY *vs.* W, Y *vs.* W, O *vs.* W, OR *vs.* W), and the sweetpotato carotenoid metabolic pathway were mapped (**Figure 3**). The pathway was constructed based on KEGG and references (Suematsu et al., 2020; Xu et al., 2021; Yuan et al., 2021; KEGG, 2022). Of which 10 *DEGs* namely *Or* (1), *CRTISO* (1), *CYP97B2* (1), *CHYB* (1), *ZEP* (2), *NSY* (1) and *DWARF27* (3) were playing major role in carotenoid biosynthesis pathway and 17 DEGs namely *CCD1* (8), *CCD4* (1), *CCD7* (1), *NCED* (4) and *CYP707A* (3) were related to carotenoid degradation pathway. The expression of 27 genes was shown in **Tables S11, S12**. Except for *CYP707A-2* gene, the expression levels of these genes varied significantly among five cultivars. Heatmap (**Figure 3**) illustrates the expression patterns of 27 DEGs. For qRT-PCR analysis, 5 key DEGs were randomly selected to validate their expression level (**Table S13**).

Pearson correlation analysis between the gene expression and the carotenoid contents was shown in **Table S14**. The expression levels of genes involved in biosynthesis and degradation of various carotenoids showed significant correlation with carotenoid content. For instance, *DWARF27-1* gene expression was significantly positively correlated with all individual carotenoids. The expression of *CCD1-2* gene was significantly negatively correlated with all individual carotenoids except for lutein and zeaxanthin palmitate. The expression of one *ZEP* gene, g1103 showed significantly negative correlation with lutein content, and the expression of the other *ZEP* gene g1106 showed significantly positive correlation with antheraxanthin content. The expression of *CCD1-6/7/8*, *CCD7*, *CHYB*, *DWARF27-2*, *NCED-2* and *NSY* genes was significantly positively correlated with the content of three carotenes. *Or* gene expression was significantly positively correlated with the content of three carotenoid esters, antheraxanthin, violaxanthin and neoxanthin. However, the expression of *NCED-1*, *NCED-4*, *CYP707A-2*, *CYP707A-3* and *CRTISO* was not significantly correlated with the content of any carotenoid compositions.

### WGCNA module analysis

**Figure 4:**
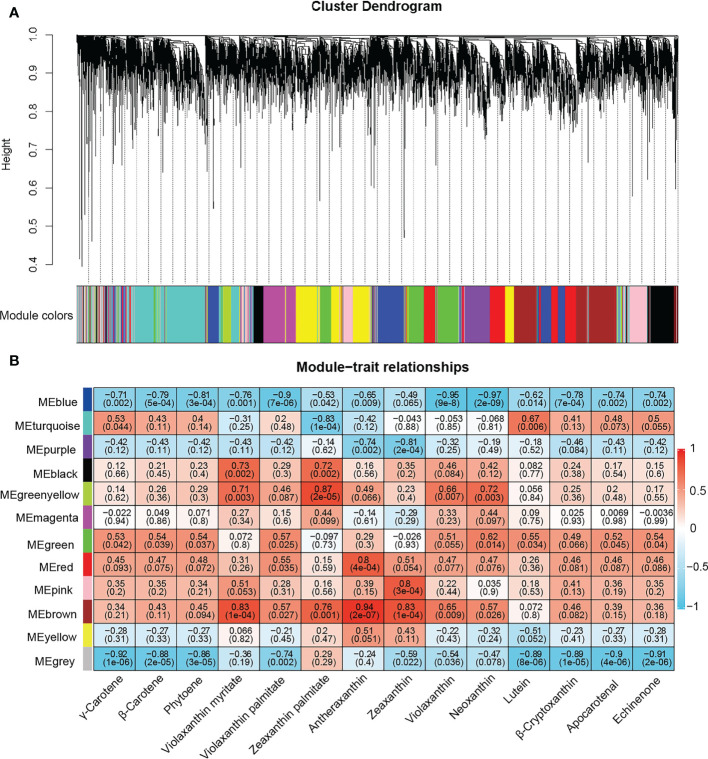
Weighted gene co-expression network analysis (WGCNA) of differentially expressed genes. **(A)** Hierarchical clustering tree showing 12 gene modules of co-expressed genes, each module labeled with different color. Each leaf in branch of the tree represented one gene. **(B)** Relationship between gene module and carotenoids contents in sweetpotato cultivars. Each row corresponds to a module, labeled with different colors. Each column represents a carotenoid component. The values in each cell represent the correlation coefficient and *P* value between the module and carotenoids and displayed according to the color scale on the right.

WGCNA is a system biology approach to describe association patterns of gene expression between different samples and can be used for finding the highly correlated genes related to phenotypes (Langfelder and Horvath, 2008). In this study, after removing 90% of the DEGs with FPKM values< 1, a total of 9,151 DEGs were retained and divided into 12 modules (**Figure 4A** and **Table S15**). The clustering heatmap of DEGs in gene modules was drawn to show the correlations between gene modules (**Figure S4**). The analysis of module-carotenoid relationships revealed that the blue and brown co-expressed modules were significantly associated with carotenoid metabolites (**Figure 4B**). The blue module containing 1,077 DEGs exhibited a highly negative correlation with carotenoid contents except for zeaxanthin with correlation coefficient (r ≤ -0.5 and *P*< 0.05), and the brown module containing 1,056 DEGs had a highly positive correlation with the content of antheraxanthin, violaxanthin myristate, zeaxanthin, zeaxanthin palmitate, violaxanthin, neoxanthin, and violaxanthin palmitate.

Heatmaps for DEGs in blue and brown modules showed two distinct clusters in each module (**Figure S5**). In blue module, the genes of cluster I showed higher expression levels in OR and O cultivars than in W, LY and Y cultivars. However, the genes of cluster II showed lower expression levels in OR and O cultivars than in W, LY and Y cultivars. Furthermore, the genes of cluster I in the brown module were highly expressed in W cultivar and the genes of cluster II showed higher expression levels in O cultivar. Therefore, we concluded that the genes in blue and brown modules must be playing different roles in the carotenoid metabolic pathways.

### Co-expression network analysis identified carotenoid related TFs

**Figure 5:**
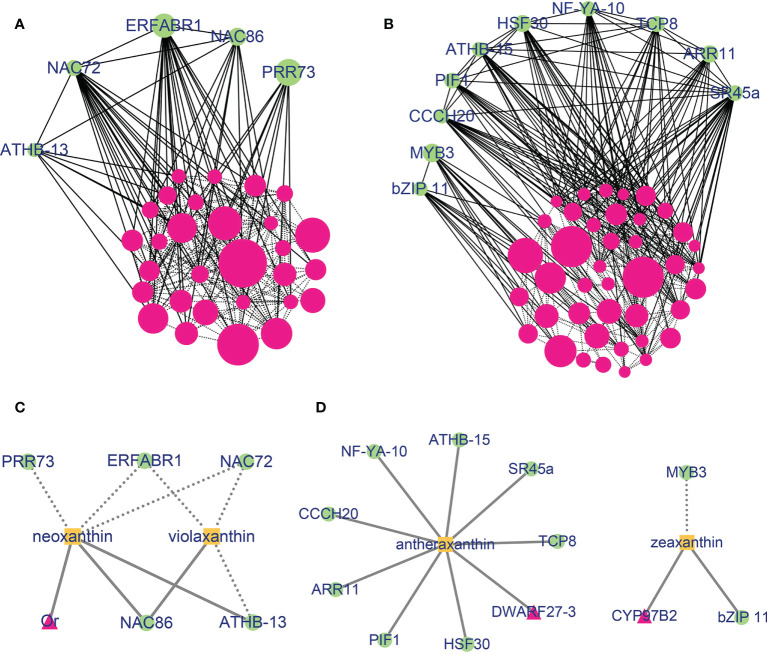
Co-expression network of genes in the blue and brown modules. **(A)** Gene co-expression network of the blue module. **(B)** Gene co-expression network of the brown module. The solid lines mean the interaction with transcription factors and dotted lines represent interaction between two genes. Dot sizes represent the weight between genes. Co-expression network of carotenoid metabolism genes and transcription factor in the blue **(C)** and brown **(D)** modules with carotenoids. The solid lines present that the genes were positively with carotenoids (GS > 0) and dotted lines represent that the genes were negatively with carotenoids (GS< 0). Rose red, proteins; Green, transcription factors; Orange, carotenoids.

To further identify transcription factors related to carotenoid content and composition and to determine the relationship between gene modules, a correlation network was constructed with edge weight ≥ 0.1 in blue and brown modules. When gene significance (GS) and eigengene connectivity (kME) was set as ≥ 0.9 or ≤ -0.9, 34 and 51 genes were used to construct a co-expression network, 5 and 10 transcription factors were identified relating to carotenoid accumulation in blue and brown modules, respectively (**Figures 5A, B** and **Table S16**). In the blue module, *NAC* transcription factor family *NAC72* and *NAC86* interact with *ERF* family member *ERFABR1* and *GATA* family member *ATHB-13.* The transcription factors *NAC72*, *ERFABR1* and *PRR73* were highly expressed in Y, and lowly expressed in OR and O cultivars, while the transcription factors *NAC86* and *ATHB-13* showed higher expression abundance in O and OR cultivars (**Figure S6**). In the brown network, transcription factor *SR45a* showed interaction with *CCCH20*, *PIF1*, *ATHB-15*, *HSF30*, *NF-YA-10*, *TCP8* and *ARR11*. Furthermore, the expression trend of *SR45a* was completely consistent with the expression pattern of *ARR11*, *TCP8*, *PIF1*, and *ATHB-15* among the five sweetpotato cultivars. Interestingly, *MYB3* interacts with *bZIP11*, but the expression pattern of the two genes was opposite. For instance, *MYB3* was highly expressed in W, while the expression of *bZIP11* was lower in W cultivar (**Figure S6**).

Additionally, we constructed the networks between differentially expressed transcription factors (DETFs) and DEGs related to carotenoid metabolic pathway in the blue and brown modules and carotenoids content based on GS to further investigate the role of hub genes in regulation of carotenoid accumulation (**Figures 5C, D** and **Table S17**). In the blue module, *NAC72* and *ERFABR1* were significantly negatively correlated with the content of neoxanthin and violaxanthin, while *NAC86* was positively correlated with these two carotenoids. In case of brown module, except for *MYB3*, the expression of DETFs and DEGs were significantly positively correlated with zeaxanthin or antheraxanthin content. These results indicated that not only the genes from blue and brown modules are correlated with the content of carotenoid, but also there are some important transcription factors that regulate the biosynthesis and accumulation of carotenoid in sweetpotato flesh.

### Validation of the expression of key DEGs by qRT-PCR

To validate the expression level of key genes related to carotenoid accumulation, we randomly selected 5 DEGs namely *CHYB*, *ZEP*, *NSY*, *NCED* and *CYP707A* for qRT-PCR. The expression of *ZEP-1*, *NCED-3* genes was higher in LY, OR, O and Y than in W cultivars, which was consistent with the result of RNA-seq. A correlation analysis exhibited a significant correlation between qRT-PCR and RNA-seq with correlation coefficient > 0.8 (**Table S13** and **Figure S7**). These results indicated that our transcriptome data is reliable.

## Discussion

### Carotenoids in different flesh-colored sweetpotatoes

Many flowers, fruits, seeds and roots exhibit various colors such as white, yellow, orange to red, which are mainly determined by the different proportions of carotenoid content (HOWITT and POGSON, 2006; Zhou et al., 2020; Xu et al., 2021). The correlation analysis between color index and carotenoid contents showed that L* and H° values were significantly negatively correlation with carotenoids contents, while a*, b*, and C* values were opposite (**Table S18**). The results showed that the color in tuberous root of sweetpotato, ranging from white, light-yellow, yellow, orange to orange-red, is also attributed to the different level and ratio of carotenoids. In this study, 14 carotenoids were identified in five sweetpotato cultivars with different flesh colors by LC-MS/MS **(** **Table 1**). The individual carotenoids contents were significantly different among five sweetpotato cultivars. The content of β-carotene and β-cryptoxanthin in W cultivar was lower, and gradually increased with the deepening of flesh color from white to orange-red, which was consistent with the findings of Alam et al. (Alam et al., 2016) and Islam et al. (Islam et al., 2016). However, β-cryptoxanthin and zeaxanthin were main carotenoids in yellow and light-yellow cultivars, β-carotene present in high proportion in orange and orange-red cultivars, which was consistent with Ishiguro’s report (Ishiguro et al., 2010). Our results indicated that the yellow and orange flesh of sweetpotatoes may be caused by the yellow xanthophylls and orange β-carotene carotenoids.

Carotenoids encompass carotenes and xanthophylls (also called carotene derivatives that contain one or more oxygen atoms) (Armstrong, 1997). What’s more, xanthophylls possessing hydroxyl groups are present in the form of free or esterified xanthophylls with different fatty acids (Mariutti and Mercadante, 2018). In present study, zeaxanthin palmitate, violaxanthin palmitate and violaxanthin myristate were identified in small amounts for the first time in sweetpotato fleshes (**Table 1**). Similar to the finding of Breithaupt and Bamedi (Breithaupt and Bamedi, 2002), violaxanthin and zeaxanthin were the main esterified carotenoids in potato fleshes, mainly esterified to the fatty acid of palmitic acid and myristic acid. Fernandez-Orozco et al. (Fernandez-Orozco et al., 2013) found that the esterified carotenoid fraction was positively correlated with the total carotenoid content in sixty potato cultivars. However, that was not consistent with the results of this study, which may be due to the low lipid content and carotenoid esters content in sweetpotatoes. These results suggest that the carotenoid esterification may be species-specific and further experiments are needed to verify. Carotenoids are present in the form of esterification in plants, which influences the interaction between carotenoids and other molecules and affects carotenoid sequestration and accumulation (Watkins and Pogson, 2020). In this study, the content of esterified carotenoid is very low, so it is not necessary to be considered in the study of the mechanism of carotenoid accumulation in sweetpotatoes.

### The key genes involved in carotenoid metabolic pathway

The mechanism of carotenoid accumulation is mainly regulated by biosynthesis, degradation, and sequestration processes (Watkins and Pogson, 2020). Currently, researchers studying carotenoid accumulation are mainly concerned with transcriptional regulation, especially those genes involved in carotenoid metabolic pathway. Studies have shown that *CRTISO* (prolycopene isomerase) is one of the enzymes that catalyze the synthesis of lycopene from phytoene, and has a critical role in carotenoid biosynthesis pathway in the dark and in non-photosynthetic tissues (Isaacson et al., 2002). Suematsu et al. (Suematsu et al., 2020) identified *ZEP* paralog (g1103.t1) as a key gene involves in carotenoid accumulation by regulating epoxidation of β-carotene and β-cryptoxanthin in yellow-fleshed sweetpotato. Cytochrome P450 (*CYP97*) family is the key enzyme that catalyzes carotene to produce xanthophylls (Li and Wei, 2020). In Arabidopsis, it was reported that *CYP97C1* hydroxylated both β- and ϵ-rings of α-carotene, while *CYP97A3* was most active on β-ring of α-carotene. Subsequent studies revealed that *CYP97B* family members were β-ring specific, similar to *CYP97A* (Niu et al., 2020). In this study, *CYP97B2* gene (g33351) encodes carotenoid epsilon-hydroxylase was identified in O *vs.* W and OR *vs.* W combinations. The function annotation of *CYP97B2* gene in sweetpotato was not consistent with the research results of Niu et al. (Niu et al., 2020), this may be due to the different carotenoid accumulation mechanism of sweetpotato and Arabidopsis. *Or* gene encodes an orange protein involved in accumulation of carotenoid in plants. Overexpression of *Or* gene has been shown to increase carotenoid content by post-transcriptionally regulating PSY (Zhou et al., 2015). In the present study, we found that the expression of *Or* gene (g20467) was significantly different between five sweetpotato cultivars, but the PSY gene showed no significant difference. This may be attributed to that PSY gene expression is not only affected by orange protein but also regulated by the other transcription factors, such as phytochrome-interaction factors (PIFs) (Toledo-Ortiz et al., 2010).

Interestingly, 27 DEGs were identified involved in the carotenoid metabolic pathways. Among these, except for *CRTISO*, *NCED-1/4* and *CYP707A-2/3*, the gene expression levels were significantly correlated with carotenoid content (**Table S14**). However, the variation of carotenoid accumulation among five sweetpotato cultivars was not completely consistent with its upstream carotenogenic genes expression trend. The *CRTISO* gene (g59407) showed different expression patterns in five sweetpotato cultivars, and that expression level was upregulated in LY and O cultivars (**Figure 3**), lycopene was not detected in any of the sweetpotato cultivars. The content of neoxanthin was higher in OR and O than in W, LY and Y cultivars, while the expression of NSY gene was significantly higher in OR and LY than in Y, O and W cultivars. Although violaxanthin and antheraxanthin were highly accumulated in OR and O cultivars, the expression level of ZEP gene was higher in LY and Y cultivars. We concluded that the accumulation of carotenoid was not directly correlated with the expression of upstream carotenogenic genes. These results are consistent with the findings of Watkins et al. (Watkins and Pogson, 2020). Therefore, the study on the mechanism of carotenoid coloration should focus on the regulation of transcription factors as well as gene transcription in carotenoid metabolic pathway.

### Transcription factor related to carotenoid metabolic pathway in sweetpotato

In recent years, several transcription factors that regulate and control the carotenoid metabolic pathway have been identified in several crop species (Ampomah-Dwamena et al., 2019; Yuan et al., 2021). In present study, we performed WGCNA to identify transcription factors that coordinately regulate the biosynthesis and accumulation of carotenoids. We found 15 differentially expressed transcription factors that were related to carotenoid content in sweetpotato fleshes. As an underground part of the plant, the tuberous root of sweetpotato is susceptible to abiotic stresses, such as water, temperature, salt and other environmental cues (Stanley and Yuan, 2019). Previous studies showed that some transcription factors were positively or negatively correlated with carotenogenic genes, and regulated the expression of abiotic stress-responsive genes in crops. The CCCH-type zinc finger protein *IbC3H18* is a key regulator of abiotic stress response in sweetpotato, such as ABA signaling, reactive oxygen species scavenging (Zhang et al., 2019). In Arabidopsis, the serine/arginine-rich splicing factor, SR45a, was found to participate in the regulation of salinity tolerance (Li et al., 2021). Huang et al. reported that the heat-stress factor *HSFA6b* participated in the positive regulation of ABA mediated salt and drought resistance (Huang et al., 2016). We found that *CCCH20*, *HSF30* and *SR45a* genes showed different expression in five sweetpotato fleshes, and were significantly positively correlated with antheraxanthin content. The downstream products of antheraxanthin, violaxanthin and neoxanthin, can be degraded by *NCED* to produce abscisic acid (ABA). We speculated that abiotic stress response of sweetpotato regulated the biosynthesis of carotenoid and these transcription factors might regulate the biosynthesis of carotenoid downstream products by participating through ABA signal transduction.

Phytochrome-interacting factors 1 (PIF1) specifically inhibited *PSY* gene by interacting with other transcription factors to regulate carotenoid synthesis (Toledo-Ortiz et al., 2010). In kiwifruit, a *R2R3-MYB* transcription factor, *MYB7* was found to regulate carotenoid accumulation by activating the promoter of the lycopene beta-cyclase (*LCYB*) gene (Ampomah-Dwamena et al., 2019). In this study, the expression of *PIF1* gene was positively correlated with the content of antheraxanthin, and the *MYB3* was negatively related to zeaxanthin content. The results indicated that *PIF1* and *MYB3* transcription factors may be activate or inhibit the expression of carotenogenic genes to regulate the biosynthesis of antheraxanthin and zeaxanthin in sweetpotatoes. Although the key genes involved in the carotenoid biosynthesis pathway and the transcription factors related to carotenoid metabolism have been identified, the regulatory mechanisms and gene functions of carotenoid coloration still need to be further investigated and verified.

The carotenoid metabolites in five sweetpotato cultivars with different flesh colors were successfully determined by LC-MS/MS. A total of 14 carotene metabolites were detected in sweetpotato fleshes, and β-carotene was obviously dominant in orange and orange-red colored fleshes, β-cryptoxanthin and zeaxanthin were enriched in yellow and light-yellow colored fleshes, while lutein was abundant in white colored flesh. Moreover, in the present study, zeaxanthin palmitate, violaxanthin palmitate and violaxanthin myristate were identified in small amounts for the first time in sweetpotato fleshes. Combined with different metabolites and transcriptome analysis, 27 enzymatic genes in carotenoid biosynthesis and degradation were identified in four sweetpotato combinations. Through WGCNA, 15 differentially expressed transcription factors were found to be significantly correlated with carotenoids content, which may be involved in carotenoid accumulation and affect the formation of sweetpotato flesh color.

## Data availability statement

The datasets presented in this study can be found in online repositories. The names of the repository/repositories and accession number(s) can be found in the article/**Supplementary Material.**

## Author contributions

Conceptualization and methodology: RJ and ZW; material provider: BJ; formal analysis: RJ and RZ; data curation: RJ; writing original draft preparation: RJ; writing review and editing: ZW, GL, RZ, SG, CT, and BJ; visualization: RJ; funding acquisition: ZW. All authors contributed to the article and approved the submitted version.

## Funding

This work was supported by National Key R&D Program of China (nos. 2019YFD1000700 and 2019YFD1000701), the earmarked fund for CARS-10-Sweetpotato and the Guangdong Modern Agro-industry Technology Research System (2022KJ111).

## Conflict of interest

The authors declare that the research was conducted in the absence of any commercial or financial relationships that could be construed as a potential conflict of interest.

## Publisher’s note

All claims expressed in this article are solely those of the authors and do not necessarily represent those of their affiliated organizations, or those of the publisher, the editors and the reviewers. Any product that may be evaluated in this article, or claim that may be made by its manufacturer, is not guaranteed or endorsed by the publisher.

## Abbreviations

GGPP, geranylgeranyl diphosphate; GGPS, pyrophosphase synthase; IPP, isopentenyl diphosphate isomerase; DMAPP, dimethylallyl diphosphate; WGCNA, weight gene co-expression network analysis; RNA-Seq, transcriptome sequencing; LC-MS/MS, metabolomics; qRT-PCR, quantitative real-time PCR; DEGs, differentially expressed genes; DETFs, differentially expressed transcription factors; FDR, false discovery rate; PCC, pearson correlation coefficient; PSY, 15-cis-phytoene synthase; PDS, 15-cis-phytoene desaturase; Z-ISO, 15-cis-ζ-carotene isomerase; ZDS, ζ-carotene desaturase; CRTISO, prolycopene isomerase; LCYB, lycopene β-cyclase; LCYE, lycopene ϵ-cyclase; DWARF27, β-carotene isomerase D27; CCD7, carotenoid cleavage dioxygenase 7; CHYB, β-carotene hydroxylase; CYP97B2, cytochrome P450 97B2-type epsilon-hydroxylase; ZEP, zeaxanthin epoxidase; VDE, violaxanthin de-epoxidase; NSY, neoxanthin synthase; NCED, 9-cis-epoxycarotenoid dioxygenase; ABA2, xanthoxin dehydrogenase; CYP707A, (+)-abscisic acid 8’-hydroxylase.
