# Supplementary material for: Integrated analysis of carotenoid metabolites and transcriptome identifies key genes controlling carotenoid compositions and content in sweetpotato tuberous roots (Ipomoea batatas L.)
Source: Front Plant Sci. 2022 Oct 20;13:993682. doi: 10.3389/fpls.2022.993682 (PMC9632283; doi:10.3389/fpls.2022.993682)
Supplement: Supplementary file 1 [file DataSheet_1.docx]

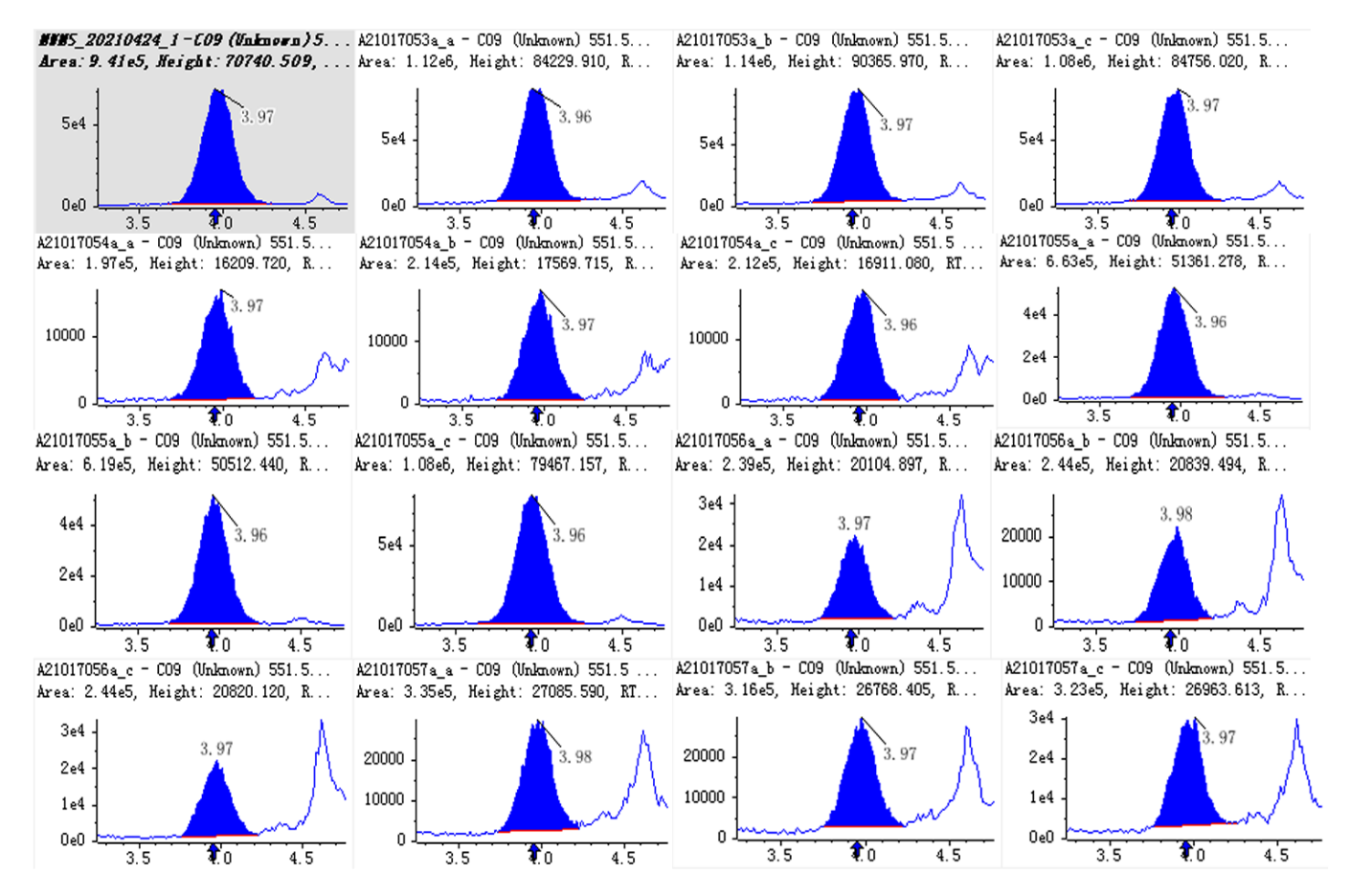


Figure S1 Integral calibration chart of C09 (lutein) in different sweet potato fleshes. The abscissa is the retention time of detection, and the ordinate is the ion current intensity of ion detection. Area and height represent the peak area and height of chromatographic peaks, respectively. MWMS_20210421_1, A21017053a, A21017054a, A21017055a, A21017056a and A21017057a represent carotenoid standard substance, OR, LY, W, Y, O, respectively.


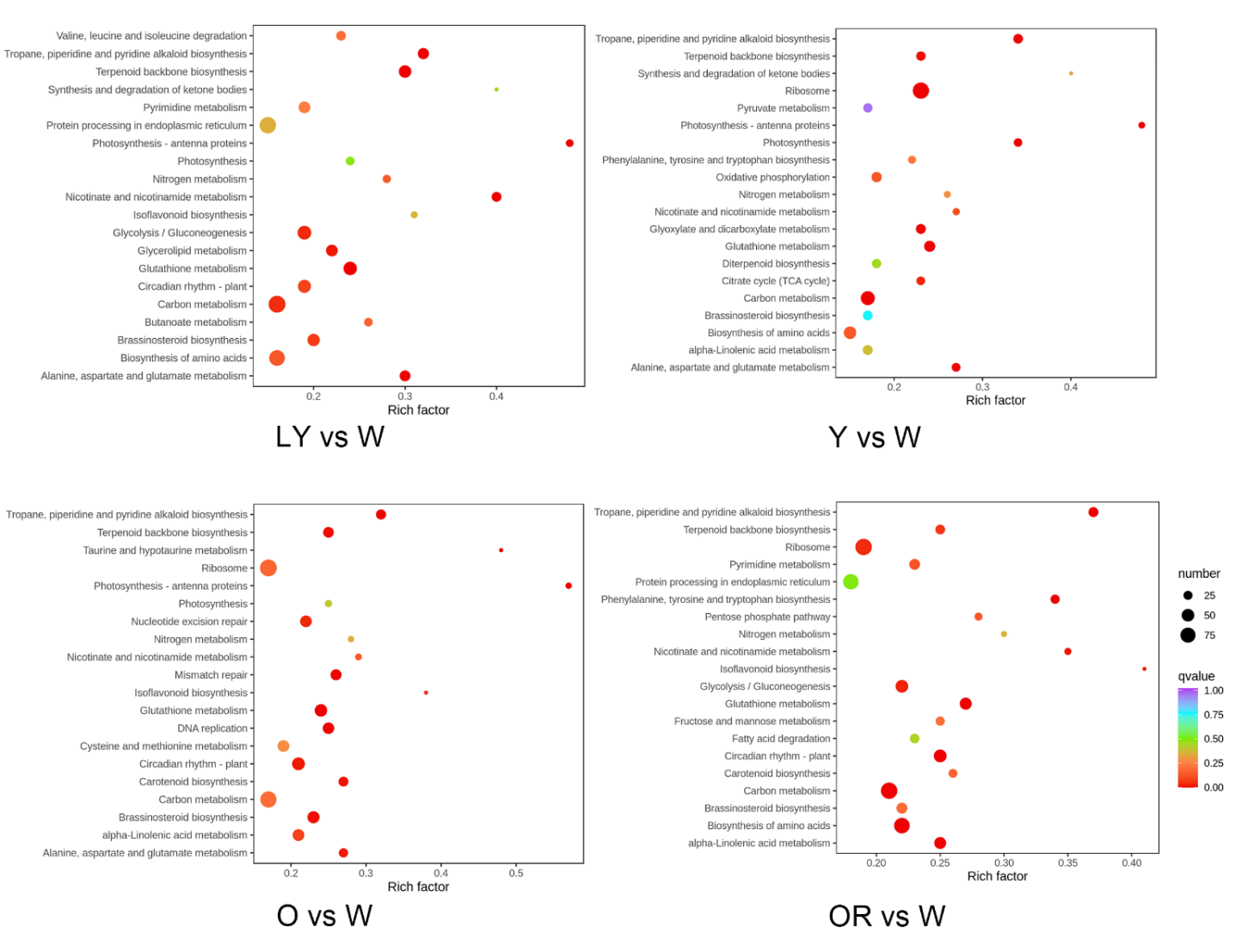


Figure S2 The top 20 significant KEGG pathway of DEGs in four groups. Shangshu 19, Okinawa-No.100, Jieshu 95-16, Guangshu 87, Pushu 32 cultivars were renamed as W, LY, Y, O, OR, respectively.


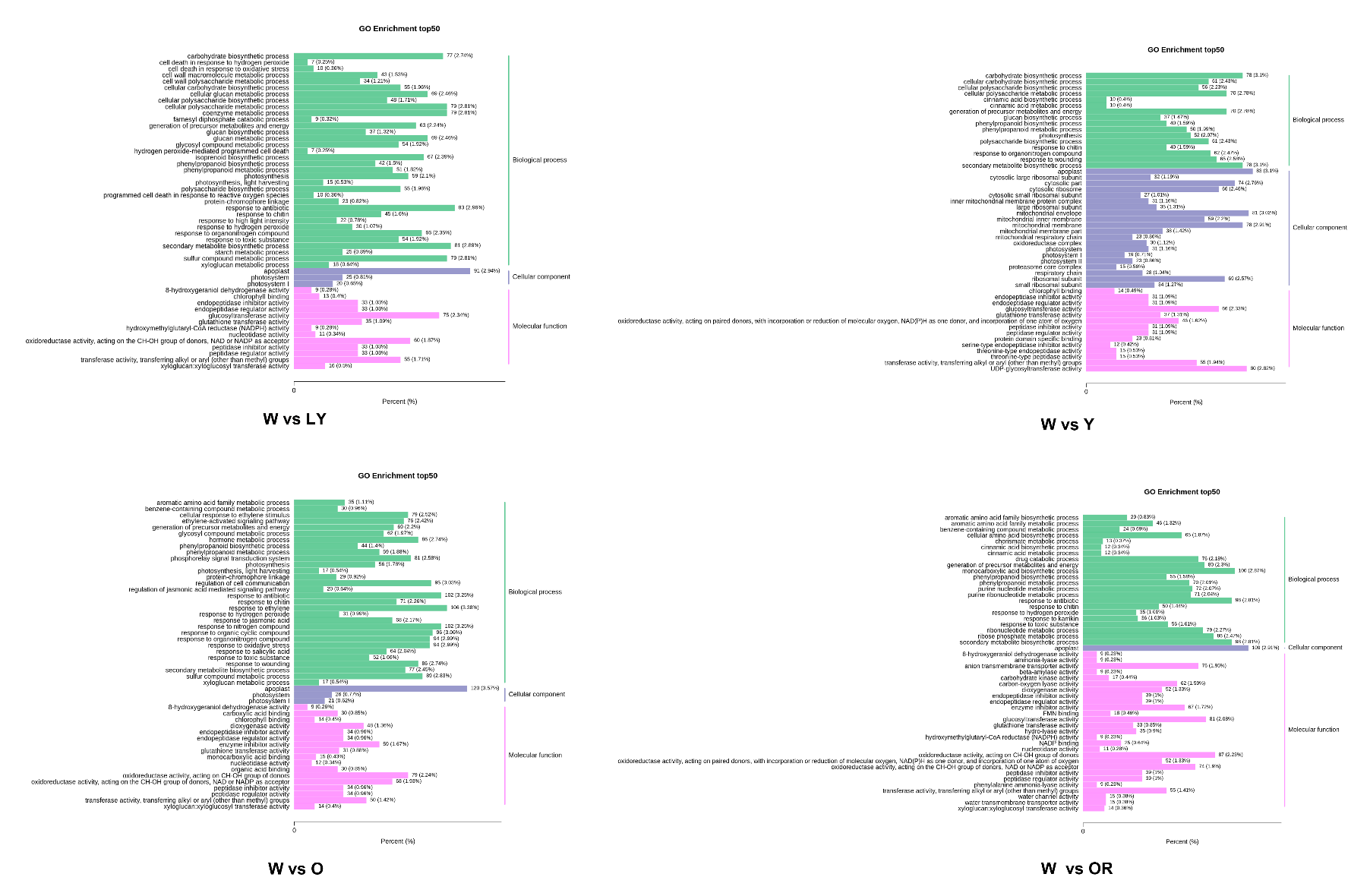


Figure S3 GO enrichment analysis of DEGs in four combinations. DEGs were annotated in three categories: biological process (green), cellular component (purple) and molecular functions (pink). X-axis indicates the number of genes in a category. Shangshu 19, Okinawa-No.100, Jieshu 95-16, Guangshu 87, Pushu 32 cultivars were renamed as W, LY, Y, O, OR, respectively.


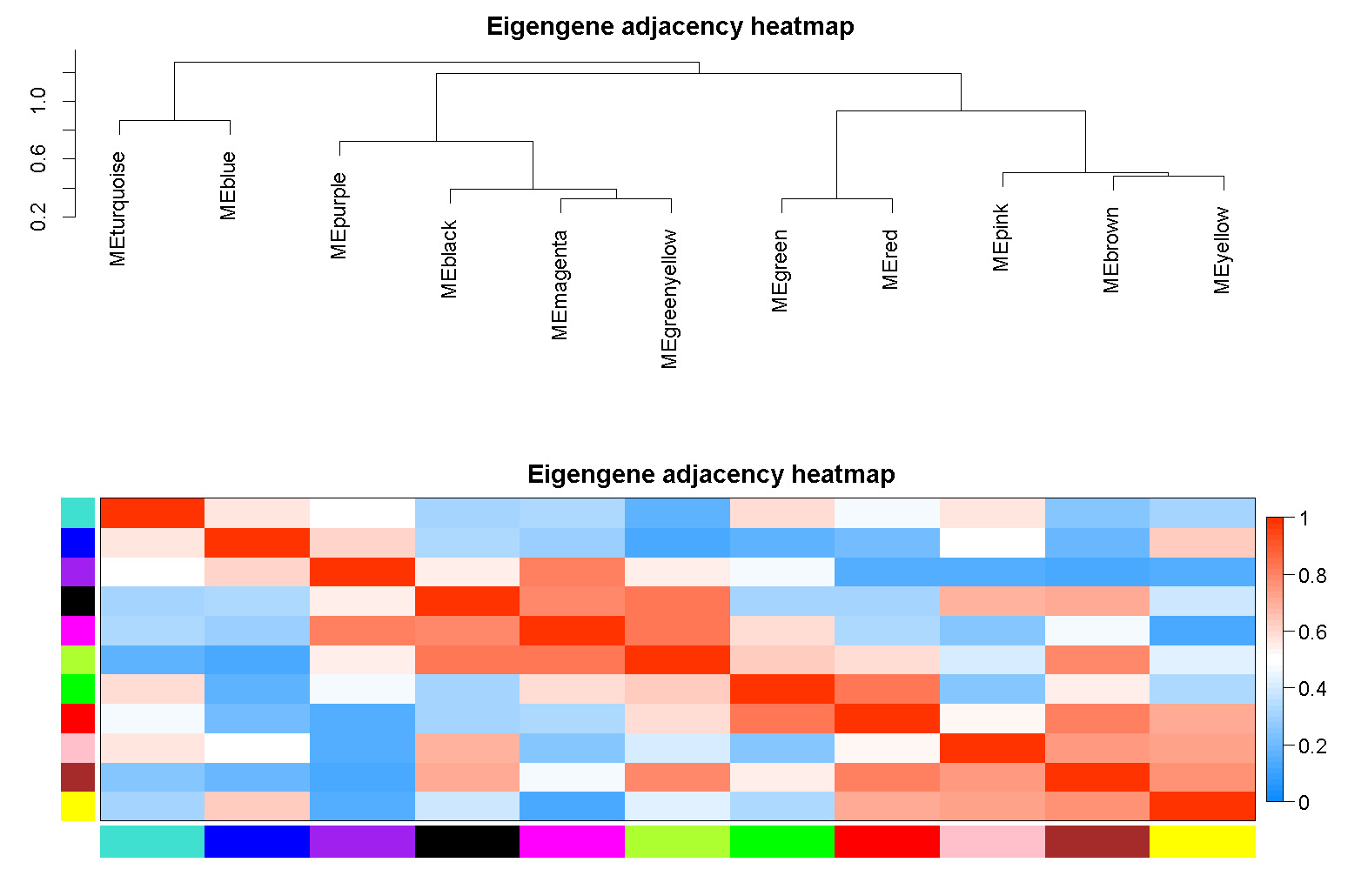


Figure S4 Heatmap and clustering of 12 gene modules


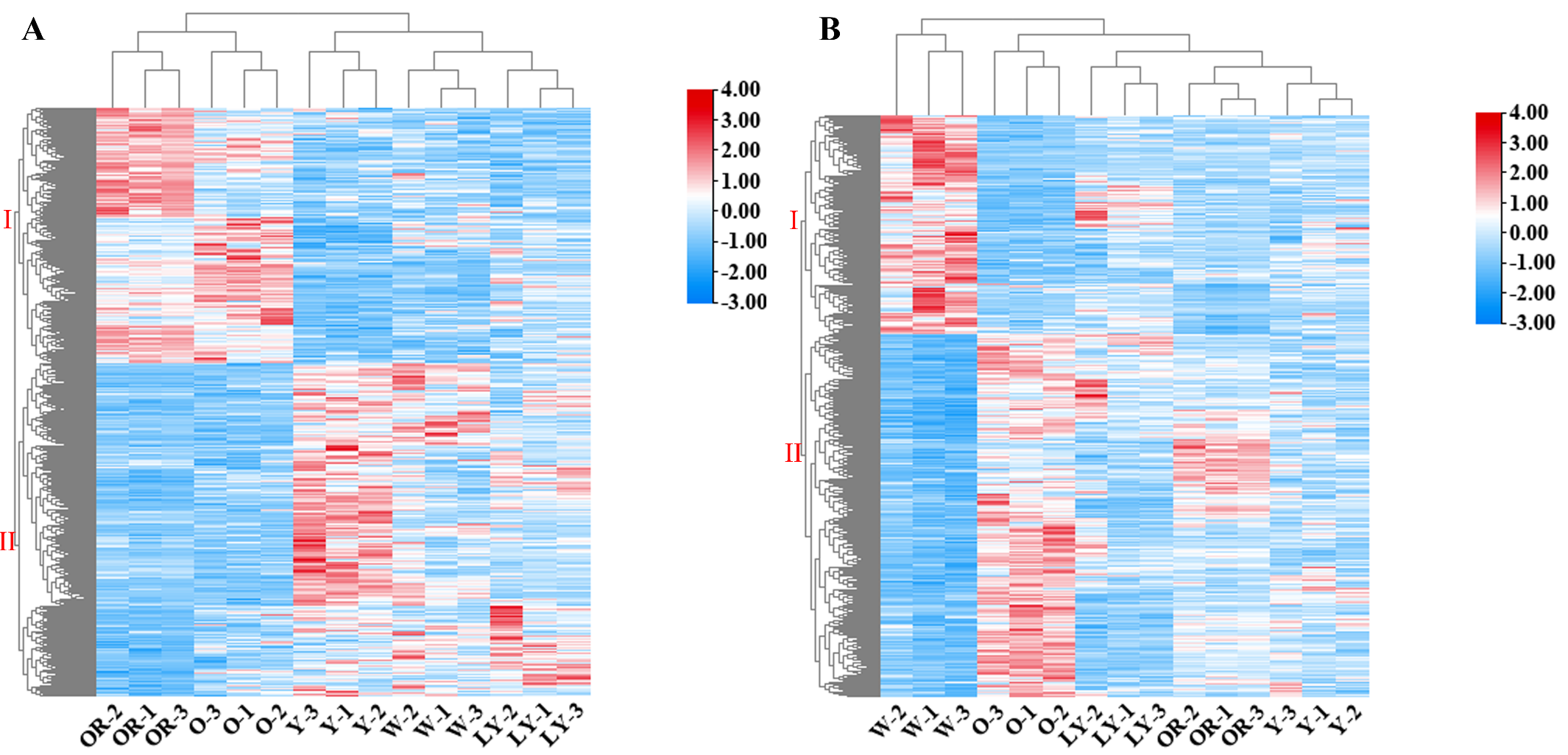


Figure S5 Heatmap illustrating the expression patterns of DEGs from A) blue gene module and B) brown gene modules. Shangshu 19, Okinawa-No.100, Jieshu 95-16, Guangshu 87, Pushu 32 cultivars were renamed as W, LY, Y, O, OR, respectively.


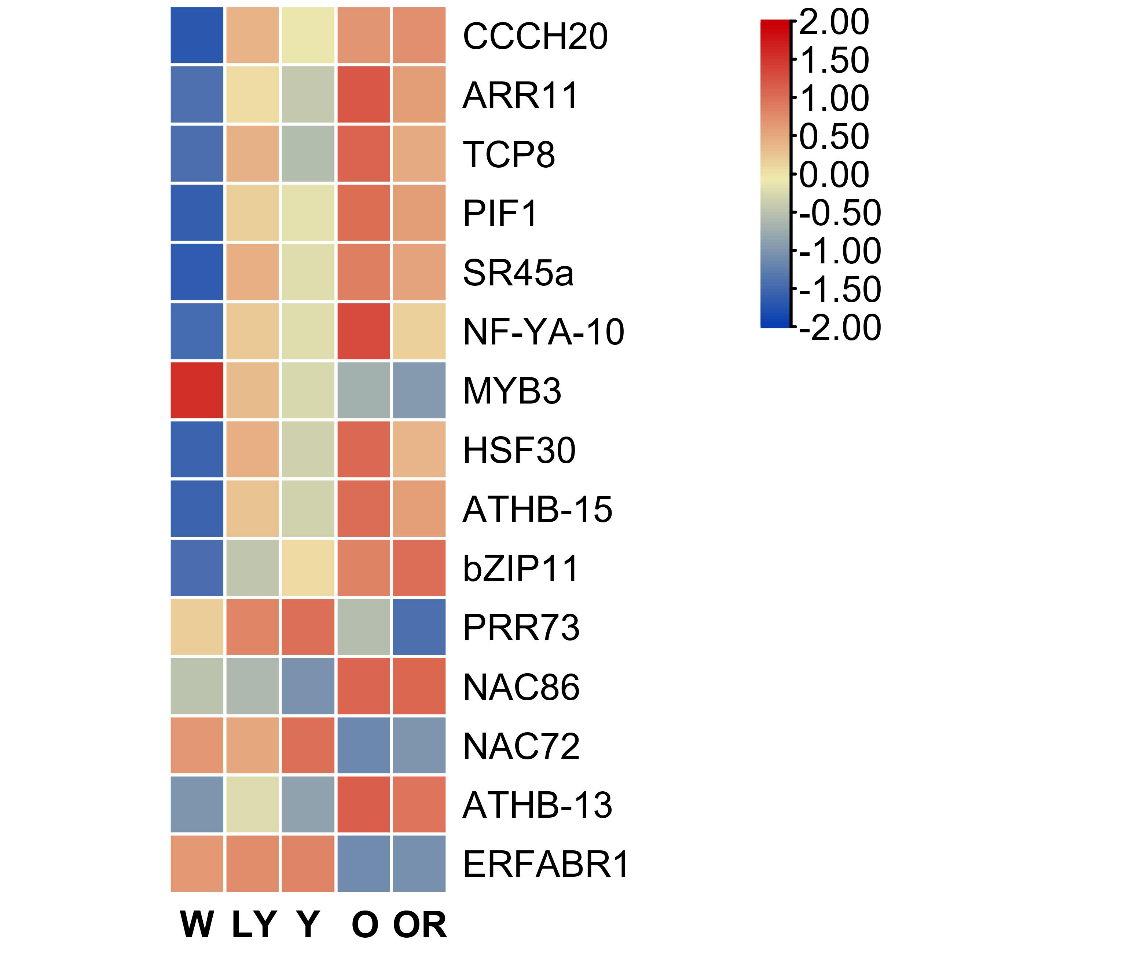


Figure S6 Heatmap illustrating the expression pattern of 15 DETFs from blue and brown modules. Shangshu 19, Okinawa-No.100, Jieshu 95-16, Guangshu 87, Pushu 32 cultivars were renamed as W, LY, Y, O, OR, respectively.


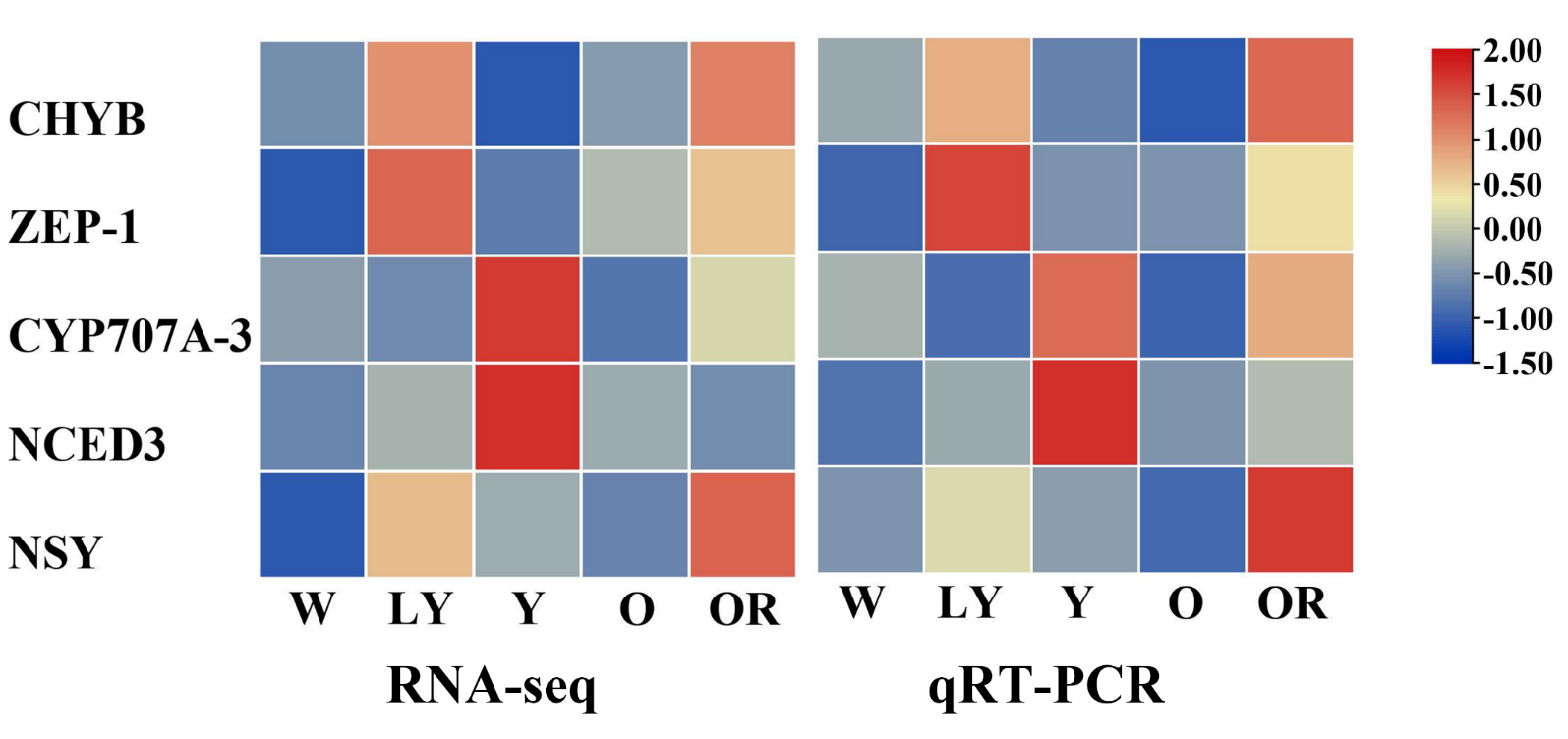


Figure S7 Heatmap illustrates the expression of the genes identified in RNA-Seq is similar to the expression values detected in qRT-PCR. Shangshu 19, Okinawa-No.100, Jieshu 95-16, Guangshu 87, Pushu 32 cultivars were renamed as W, LY, Y, O, OR, respectively. CHYB, β-carotenoid hydroxylase; ZEP, zeaxanthin epoxidase; CYP707A, (+)-abscisic acid 8'-hydroxylase; NCED, 9-cis-epoxycarotenoid dioxygenase; NSY, neoxanthin synthase.
